# Multimodal MRI Reveals Cerebral and Vascular Amyloid‐Driven Myeloarchitectural Disorganization in a Mouse Model of Alzheimer's Disease

**DOI:** 10.1002/nbm.70262

**Published:** 2026-03-13

**Authors:** Syed Salman Shahid, Xuan Li, Mario Dzemidzic, Erin E. Jarvis, Yomna Takieldeen, Aibo Wang, Donna Wilcock, Yu‐Chien Wu

**Affiliations:** ^1^ Department of Radiology and Imaging Sciences Indiana University School of Medicine Indianapolis Indiana USA; ^2^ Indiana Alzheimer's Disease Research Center Indiana University School of Medicine Indianapolis Indiana USA; ^3^ Center for Neuroimaging, Department of Radiology and Imaging Sciences Indiana University School of Medicine Indianapolis Indiana USA; ^4^ Department of Neurology Indiana University School of Medicine Indianapolis Indiana USA; ^5^ Stark Neuroscience Research Institute Indiana University School of Medicine Indianapolis Indiana USA; ^6^ Center for Neurodegenerative Disorders Indiana University School of Medicine Indianapolis Indiana USA; ^7^ Weldon School of Biomedical Engineering at Purdue University Indiana USA

## Abstract

Alzheimer's disease (AD) is a multifactorial neurodegenerative disorder involving a complex interaction of cerebral and vascular amyloid‐beta (Aβ) accumulation, myelin disruption, lipid alterations, and cerebrovascular dysfunction. The early detection and differentiation of these interconnected pathologies remain challenging. To understand the effect of cerebral and vascular Aβ on regional myeloarchitecture and lipid composition, we developed a novel multimodal neuroimaging approach integrating quantitative MRI (qMRI), chemical exchange saturation transfer (CEST) MRI, and immunohistochemistry (IHC). The framework was applied to a 10‐month‐old mouse model exhibiting both cerebral and vascular amyloid pathologies. High‐resolution in vivo MRI was performed using a 9.4 Tesla scanner. The results suggest region‐specific vulnerability to Aβ pathology with significant regional increases in apparent transverse relaxation rate (R2*, *p* < 0.05; Hedges' *g* = 1.22) and quantitative susceptibility mapping (*χ*, *p* < 0.01; Hedges' *g* = 1.99) within the hippocampus of ARTE10 mice compared to wild‐type littermates. Multislice CEST‐based Z‐spectra were used with multipool Lorentzian fitting and quantitative T1 longitudinal relaxation maps to obtain nuclear Overhauser enhancement (NOE) weighted apparent exchange‐dependent relaxation (AREX) maps. NOE _(−3.5 ppm)_‐sensitive CEST imaging contrast showed region‐specific changes in the hippocampus (*p* < 0.01; Hedges' *g* = −1.81), corpus callosum (*p* < 0.01; Hedges' *g* = −2.39), and thalamus (*p* < 0.01; Hedges' *g* = −2.64) of ARTE10 animals relative to WT littermates. Hippocampal Aβ burden, iron load, and myelin density were quantified using immunohistochemistry, suggesting strong Aβ plaque presence and elevated iron load in the hippocampi of ARTE10 mice compared to WT mice. Collectively, our results demonstrate the utility of this multimodal MRI framework in identifying sensitive and specific biomarkers of amyloid‐driven myeloarchitectural and molecular changes. The proposed framework offers a valuable tool for enhancing early detection, understanding different pathophysiological pathways, and facilitating therapeutic monitoring and targeted intervention strategies.

## Graphical abstract

This study investigates the effect of cerebral and vascular (beta‐amyloid) Aβ on regional myeloarchitecture and lipid composition in a murine model of Alzheimer's disease (AD). The study highlights the feasibility of quantitative MRI and chemical exchange saturation transfer (CEST) imaging to identify multiple biophysical mechanisms involved in pathophysiology of AD in vivo.

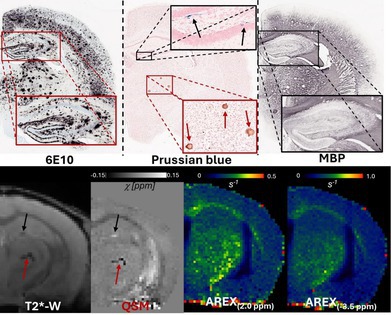

## 1 Introduction

Alzheimer's disease (AD) is a heterogeneous neurodegenerative disorder characterized by cognitive deficit and behavioral alterations. Despite immense efforts on different fronts, the disease's etiology and pathophysiology are not fully understood, and so far, there is no tangible cure for dementia. AD is a complex and multifactorial disease involving the accumulation of extracellular amyloid‐beta (Aβ) plaques, intracellular neurofibrillary tangles, accompanied by inflammation and mitochondrial dysfunction [1]. Additionally, cerebrovascular dysfunction has also been implicated in the etiology of AD, suggesting a possible bidirectional relationship between vascular pathology and AD [2, 3, 4, 5].

The region‐specific abnormal depositions of Aβ and tau proteins underscore the susceptibility of the hippocampus and basal forebrain to neurodegeneration and subsequent functional deficit. The involvement of Aβ during the early stages of AD and cerebral amyloid angiopathy (CAA) instigates multiple dysregulatory mechanisms, significantly altering regional neurochemistry and macromolecular composition [4, 6]. At the mesoscale, AD‐associated processes have been implicated in cyto‐ and myeloarchitectural disorganization, perhaps due to myelin impairment and excessive iron deposition [7, 8, 9, 10]. Additionally, Aβ‐mediated cerebrovascular dysfunction is also characterized by reduced vascular reactivity and presence of cerebral microbleeds/iron deposition [5, 11, 12].

Noninvasive, nonionizing neuroimaging biomarkers that can detect these early molecular and microstructural alterations may play an important role in the early detection, monitoring therapies, and guide drug development. MR‐based molecular imaging methods such as magnetization transfer (MT) and chemical exchange saturation transfer (CEST) have successfully quantified multiple metabolic processes–associated with synaptic health [13, 14, 15, 16, 17], AD‐associated proteinopathies, neuroinflammation, and pathology‐specific neurometabolite changes [13, 18, 19, 20, 21, 22, 23, 24, 25, 26]. Recent studies have highlighted the efficacy of amide proton transfer (APT) and relayed nuclear Overhauser effect (rNOE) CEST at −3.5 ppm in detecting lipid and myelin alterations, suggesting its utility as a marker of myeloarchitectural integrity [27, 28]. Similarly, quantitative MRI (qMRI) techniques such as multiecho Multicomponent Driven Equilibrium Single Pulse Observation of T1 (MC‐DESPOT1) provide metrics like longitudinal recovery rate (R1), apparent transverse relaxation rate (R2*), and quantitative susceptibility mapping (QSM). These quantitative measures are known to be sensitive to aging‐ and AD‐associated changes in tissue relaxivity, myelin composition, and heme‐iron load [29, 30]. However, these qMRI measures suffer from nonspecificity and partial volume contamination, making it difficult to separate localized hemosiderin deposits (paramagnetic) from diamagnetic substrate such as myelin debris and/or localized Aβ deposits [31].

Given the crucial role of cerebral iron in myelin synthesis and neurotransmitter metabolism, disturbed iron homeostasis has been increasingly implicated in accelerating AD pathology through enhanced oxidative stress and neuronal insult [30, 32, 33, 34]. However, the role of oxidative stress on phospholipid composition in the presence of cerebral and vascular Aβ load remains poorly understood [35]. To elucidate these interactions, we used an ARTE10 mouse model of AD. This model exhibits both cerebral and vascular amyloid pathology, making it suitable to investigate the relationship between regional Aβ deposition, myelin/lipid composition changes, and vascular dysfunction such as iron‐induced cerebral microbleeds. In this study, we investigated how Aβ accumulation impacts myeloarchitecture, lipid/macromolecular composition, and iron load in the hippocampus, corpus callosum, thalamus, and striatum. We employed a multimodal MRI approach that integrated 2D multislice CEST MRI for lipid‐sensitive rNOE _(−3.5 ppm)_ contrast and 3D MC‐DESPOT1 imaging to quantify T1, R2*, and QSM. Immunohistochemical staining (IHC) targeting Aβ plaques (6E10), iron (Prussian Blue), and myelin basic protein (MBP) provided additional validation of our MRI findings.

## 2 Material and Methods

### 2.1 Animal Model

Male ARTE10 animals (B6.CBA‐Tg (Thy1‐PSEN1*M146V, ‐APP*Swe)) were purchased from Taconic Biosciences Inc. (Germantown, NY, USA). ARTE10 model is a double transgenic mouse model of AD that expresses mutant forms of human APP and PSEN1 [36]. This model exhibits robust plaque pathology, initially affecting anterior neocortex and subiculum, and later spreading to hippocampus and amygdala [37, 38]. Amyloid plaques also accumulate in intracortical and leptomeningeal blood vessels. Plaques are composed of both Aβ40 and Aβ42. This model shows glial inflammation associated with Aβ pathology close to 5 months and no significant tau pathology up to 20 months [36]. The model also exhibits decrease in synaptic proteins by 3–4 months and neuronal loss with significant (20%) reduction in dendritic arbor in the hippocampus by 12 months [36, 39]. Memory deficits, based on object‐recognition task and the Morris water maze, appear at 12 months of age [36]. For this study, six 10‐month‐old ARTE10 mice and six wild type (WT, C57BL/6NT) littermates were used. Ten‐month timepoint approximates the human preclinical AD stage, where amyloid accumulation and downstream pathological cascades and compensatory mechanisms are underway but cognitive/behavioral symptoms have not yet manifested. All animal care and studies were performed in accordance with the National Institutes of Health guidelines. All study procedures were approved by the Indiana University Institutional Animal Care and Use Committee.

### 2.2 Anesthesia Protocol

For MRI experiments, animals were initially anesthetized in an induction chamber under 3% isoflurane at 1 L per minute in 100% oxygen. The anesthetized mice were then transferred to an MR compatible cradle and positioned in an MRI compatible head holder to minimize head motion. Anesthesia was subsequently maintained at 1.5% isoflurane in 100% oxygen throughout imaging. Respiration rate was monitored using a pressure pad placed under the animal's abdomen, and animal body temperature was maintained by a warming pad (37°C) placed under the animal. Respiration rate was maintained between 80 and 125 breaths per minute using manual adjustments to the isoflurane vaporizer.

### 2.3 In Vivo MR Acquisition

The in vivo imaging was conducted on a horizontal 9.4T Biospec preclinical MRI system (Bruker BioSpin MRI GmbH, Germany) equipped with shielded gradients (maximum gradient strength = 660 mT/m, rise time = 4750 T/m/s). We used an 86‐mm quadrature volume resonator for transmission and a four‐element array cryo‐coil for signal reception (cryoprobe, Bruker, BioSipn). A FLASH‐based 2D anatomical reference image was acquired to facilitate the FOV planning. 3D multiecho GRE data acquisition parameters were: FOV = 16 × 16 × 4 mm^3^, TE/TR = 2.177/33.57 ms, ΔTE = 2.47 ms, eight echoes with monopolar readout gradients, two flip angles (FA = 15° and 40°), matrix size = 128 × 128 × 16, averages = 6, acquisition time (acq time) = 7 min, 10 s per FA. CEST acquisition based on a 2D turbo Rapid Acquisition Relaxation Enhancement (RARE) sequence used the following acquisition parameters: FOV = 16 × 16 mm^2^, TE/TR = 5.6/16425 ms, echo spacing = 5.6 ms, slice thickness = 0.25 mm, slice gap = 0.25 mm, number of slices = 8, matrix size = 128 × 128, RARE factor = 64, centric phase encoding order, fat suppression on, number of averages = 1, and acq time = 14 min, 50 s. Forty‐three saturation frequency offsets (−8 ppm to 8 ppm; offset step size = 0.25 ppm), and a reference offset at −300 ppm. Ten additional saturation frequency offsets (±200, ±100, ±50, ±25, ±16) were introduced to improve MT baseline estimation. For each offset, we applied two continuous‐wave (CW) radiofrequency (RF) saturation pulses (SPs) as proposed by Villano, Romdhane [40]. The amplitudes (B1_sat_) and durations (*T*_sat_) of the primary (B1_sat_ = 1 μT; block pulse shape [bp], *T*_sat_ = 5000 ms) and secondary (B1_sat_ = 1 μT; bp, *T*_sat_ = 1000 ms) saturation pulses were optimized using numerical simulations and test scans. For water saturation shift referencing (WASSR) acquisition, the geometric parameters were identical to CEST protocol with the following main acquisition parameters: TE/TR = 5.6/16425 ms, 41 saturation frequency offsets (−1.0 ppm to 1.0 ppm; offset step size = 0.05 ppm). The primary and secondary SPs had identical RF profile (B1_sat_ = 0.1 μT (bp), *T*_sat_ = 1000 ms) and acq time = 8 min, 42 s.

### 2.4 MRI Data Processing

The 3D multichannel multiecho gradient‐echo (ME‐GRE) data were coil‐combined for each FA. Coil‐combined magnitude images were calculated using the root mean square (RMS) of the channel values [41], while coil‐combined phase images were produced using the phase difference approach [42]. Apparent transverse relaxation rate (R2*) quantitative maps were created by applying the auto‐regression on linear operations algorithm [43]. The RMS‐based echo‐combined magnitude images (T2*wTE‐weighted) were created using the weighted combination of the multiecho images. The echo dependent weighting factor ω_*i*_ is given by equation 1 [44]:

(1) $$\omega_{i}={\mathrm{TE}}_{i}^{2}.S_{i}^{2}$$

where *i* = 1–8, and *S*_*i*_ is the coil combined magnitude image for the *i* ^th^ echo. The TE‐weighted magnitude T2*W image is given by equation 2:

(2) $$T2^{*}W_{\mathrm{TEw}}=\frac{\sum \omega_{i}S_{i}}{\sum \omega_{i}}$$

**FIGURE 1:**
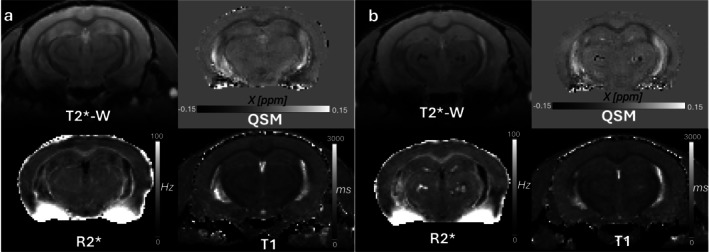
Anatomical and quantitative (qMRI) maps from a representative 10‐month‐old (a) WT and (b) ARTE10 animal. In ARTE10, hippocampal and thalamic hypointensities and hyperintensities are evident on structural T2*‐W and R2*, respectively. Differences between paramagnetic (microbleeds) in the hippocampus and diamagnetic substrate (congophilic dense‐core plaques) in the thalamus are identifiable from QSM. Wild‐type mice lack any visible pathology.

For QSM and R2* maps, we applied an initial mask using the TE‐weighted magnitude T2*W image (T2*W_TEw_). The brain mask was created using the STAPLE algorithm [45]. The skull stripped T2*W_TEw_ was corrected for B1 field inhomogeneity using N4 bias field correction algorithm [46] and then nonlinearly registered to Badhwar hippocampal atlas using symmetric diffeomorphic image registration with cross‐correlation (SyN) algorithm implemented in ANTs [47, 48]. The resulting deformation maps and transformation matrix were used to map hippocampus, corpus callosum, thalamus, and striatum masks from template space to animal‐specific T2*W_TEw_ image space. For QSM, nonlinear field fitting estimated field maps from individual FA coil‐combined complex ME‐GRE data [49] and remaining wraps were removed using Laplacian phase unwrapping [50]. V‐SHARP (SHARP with variable kernel size) was used for background field removal [51, 52]. The threshold for SHARP and V‐SHARP and radius for SHARP was evaluated on multiple datasets before batch processing. For SHARP and V‐SHARP, a boundary erosion of three voxels was performed. To avoid T1 contamination, the final QSM (*χ*) maps for FA = 15° were created using QSM iLSQR [53]. To calculate quantitative T1 longitudinal relaxation (qT1) map, we extended the DESPOT1 method [54] to multiecho data [30]. Briefly, voxel‐wise T1 values were calculated for each of the eight echoes from 15° and 40° ME‐GRE acquisitions using the DESPOT1 method, and then a weighted average of the estimated T1 map across the eight echoes was obtained (Figure 1).

For CEST imaging, voxel‐by‐voxel Z‐spectra were generated by mapping the longitudinal magnetization as a function of saturation frequency. For CEST data processing, the initial step included B0 field inhomogeneity correction using the water shift referencing method [55]. The B0‐corrected CEST data were then normalized using the reference (nonsaturated; −300 ppm) image. For CEST quantification, we used a 5‐pool Lorentzian model consisting of direct saturation (DS), MT, amines, amides, and NOE (_−3.5 ppm_) pools. In this model, DS, amines, amides, and NOE_(−3.5ppm)_ were modeled using Lorentzian line shapes as shown by Equation (3) [17, 56]:

(3) $$L\left(∆\omega_{i},A_{i,}\sigma_{i}\right)=\sum_{i=1}^{N}\left[\frac{A_{i}}{1+4{\left(\frac{∆\omega -∆\omega_{i}}{\sigma_{i}}\right)}^{2}}\right]=1-\frac{I\left(∆\omega \right)}{I_{0}}$$

where *Δω* is the frequency offset in ppm with respect to water resonance at 0 ppm, *A*_*i*_, *Δω*_*i*_, and *σ*_*i*_ are the amplitude, frequency offset, and the linewidth of the CEST peak of the *i* ^*th*^ proton pool, respectively.

The MT baseline effect was modeled using super‐Lorentzian line shape as shown by Equation (4) [57]:

(4) $$\mathrm{SL}\left(∆\omega_{\mathrm{SL}},T_{2}^{\mathrm{MT}}A_{\mathrm{SL}}\right)=A_{\mathrm{SL}}\int_{0}^{\frac{\pi }{2}}\text{dθsi}n\theta \sqrt{\frac{2}{\pi }\;}\frac{T_{2}^{\mathrm{MT}}}{\left|3\cos^{2}\theta -1\right|}e^{-2{\left(\frac{\left(∆\omega -∆\omega_{\mathrm{SL}}\right)T_{2}^{\mathrm{MT}}}{\left|3\cos^{2}\theta -1\right|}\right)}^{2}}$$

where *Δω*_*SL*_ is the frequency offset with respect to water resonance of 0 ppm, $T_{2}^{\mathrm{MT}}$ is the T2 of the MT pool, and *A*_*SL*_ is the amplitude of the MT curve. To avoid cross‐contamination from amide, amine, and NOE signals, the voxel‐wise MT contribution was estimated using Z‐spectra data points outside ±8‐ppm range. We used cubic‐spline interpolation to assess super‐Lorentzian behavior for datapoints between ±8 ppm. Additional details of Z‐spectrum fitting are available in [57]. In brief, the general equation for the normalized n‐pool Z‐spectra is given by Equation (5) [57]:

(5) $$Z_{\text{CORR}}\left(∆\omega \right)=1-L\left(∆\omega_{i},A_{i,}\sigma_{i}\right)-\mathrm{SL}\left(∆\omega_{\mathrm{SL}},T_{2}^{\mathrm{MT}}A_{\mathrm{SL}}\right)$$

The chemical shift frequencies for each proton pool, the initial conditions, and the upper and lower bounds of the fits of respective species' amplitude and linewidth parameters are described in Table 1. The Z‐spectra fitting for the five‐pool model was performed using the nonlinear fitting “*lsqnonlin*” Matlab function and the computation code was adapted from [57] to account for multislice fitting.

**TABLE 1:** Starting points and boundaries of the amplitude (*A*), peak width (*σ* in ppm), and frequency offset (Δ*ω* in ppm) of the coupling pools in the Lorentzian/super‐Lorentzian fit. The values were taken from [56, 58, 59, 60].

Pool	*A*	*σ* (ppm)/T2 (μsec)	Δ*ω* (ppm)
	LB/UB/SV	LB/UB/SV	LB/UB/SV
Water	0.02/1/0.9	0.3/10/1.4	−1/1/0
NOE_‐3.5_	0/0.6/0.02	0.5/10/3	−4/0/−2
MT	0/1/0.1	T_2_^MT^: (1/50/15) μsec	−4/4/−2
Amide	0/2/0.025	0.4/4/0.5	3/4/3.5
Amine	0/0.2/0.01	0.5/5/1.5	1.75/2.25/2.0

Abbreviations: LB, lower bound; MT, magnetization transfer; NOE, nuclear Overhauser enhancement; SV, starting value; UB, upper bound.

**FIGURE 2:**
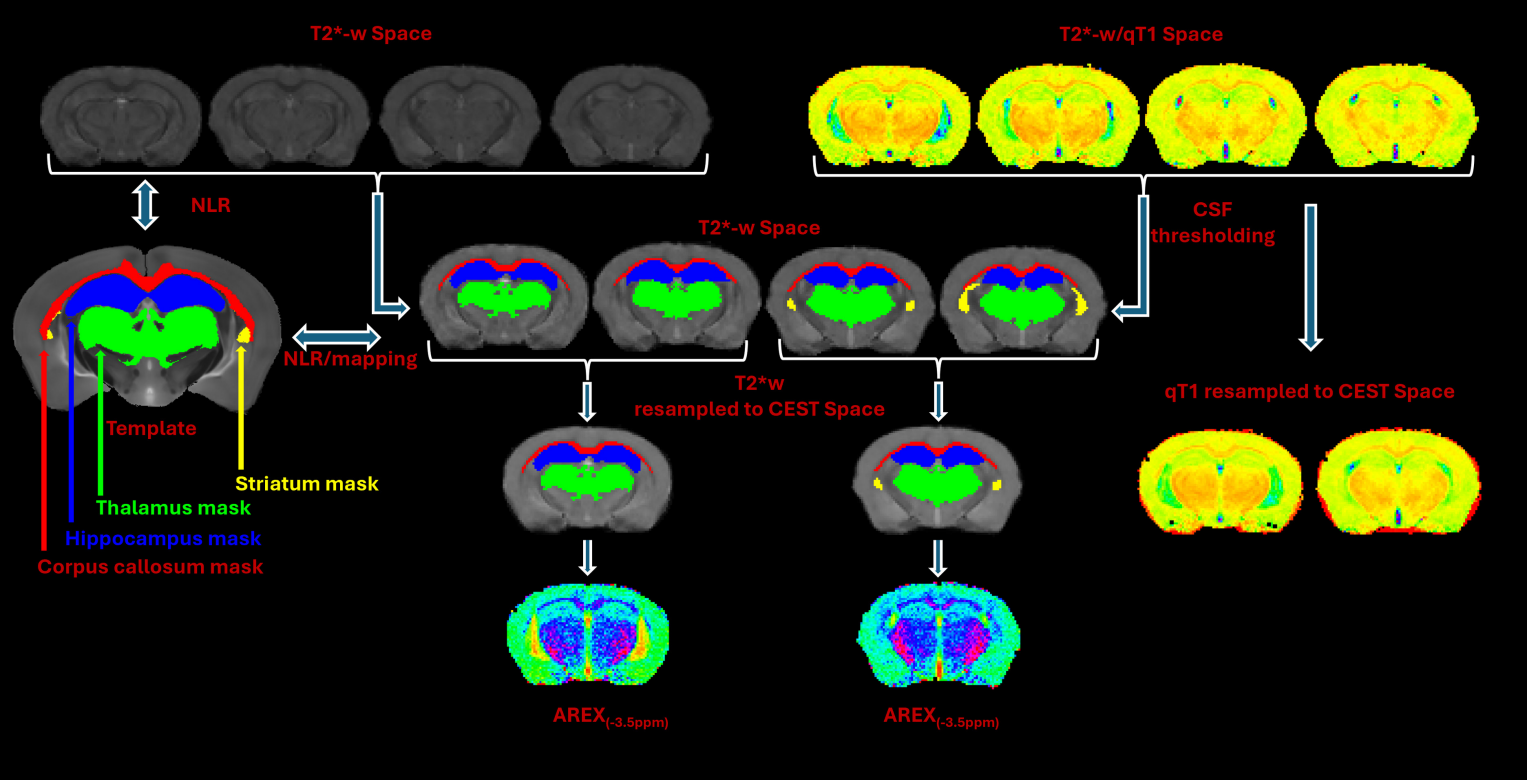
Schematic representation of the 3D qMRI and multislice 2D‐CEST data fusion workflow. AREX, apparent exchange‐dependent relaxation; NLR, nonlinear registration; ppm, parts per million.

**FIGURE 3:**
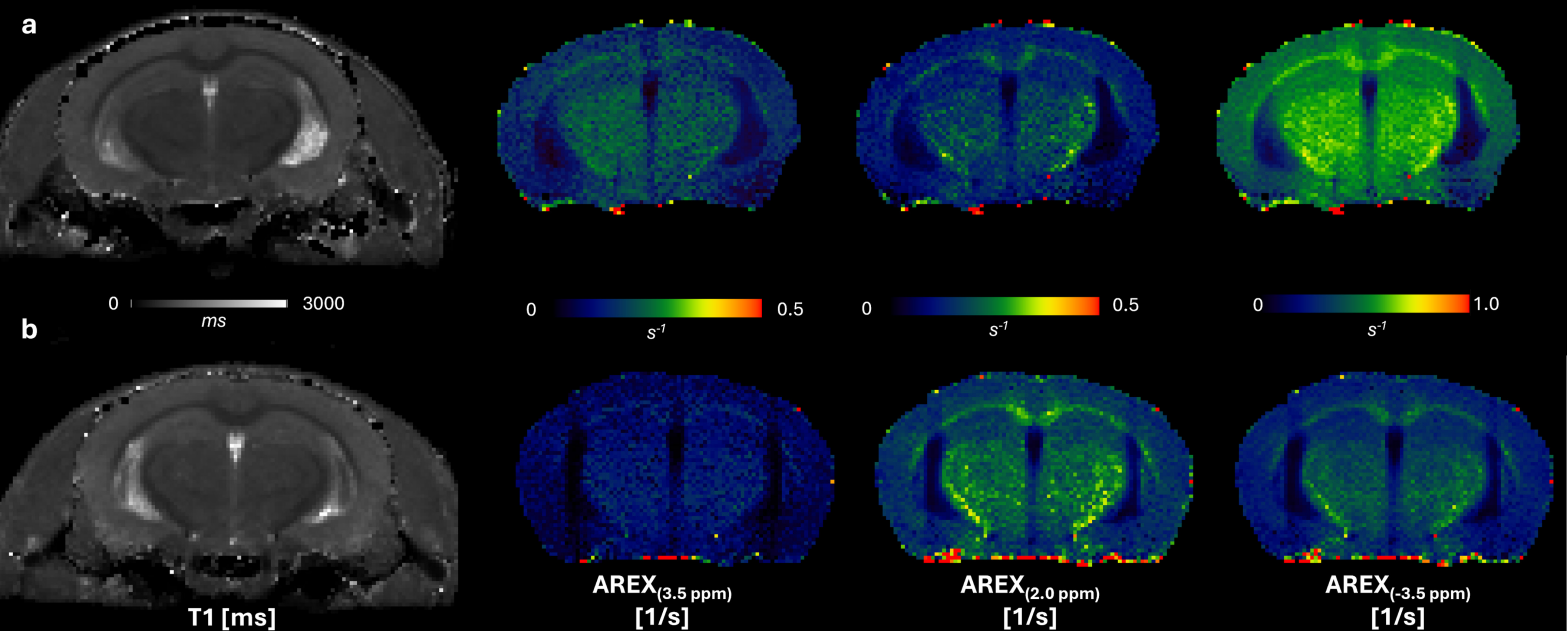
Resampled qT1 maps and CEST‐derived AREX maps at saturation frequency offsets of 3.5, 2.0, and −3.5 ppm from a representative 10‐month‐old (a) WT and (b) ARTE10 animal. AREX, apparent exchange‐dependent relaxation; ppm, parts per million.

To account for T1 effects, the estimated qT1 maps along with region of interest (ROI) masks were first downsampled to match CEST imaging resolution and then the down‐sampled qT1 was used in apparent exchange‐dependent relaxation (AREX)‐based quantification [61, 62] (Figure 2):

(6) $${\text{AREX}}_{∆\omega }=\left(\frac{1}{Z_{\text{CORR}}\left(∆\omega \right)}-\frac{1}{Z_{\mathrm{Ref}}\left(∆\omega \right)}\right).\frac{1}{T_{1}}$$

where $Z_{\mathrm{Ref}}\left(∆\omega \right)$ represents the saturation effect without the pool of interest, and $Z_{\text{CORR}}\left(∆\omega \right)$ is the complete fit of the Z‐spectra at Δω. CEST‐derived AREX maps are shown in Figures 2 and 3.

Mean ROI values of R2*, QSM (*χ*), qT1, and CEST‐derived parameters were calculated using FSL “*fslstats*” tool. To obtain robust regional mean values, individual qT1 maps were scaled at 3000 ms and then binarized to generate a CSF mask to mitigate CSF partial volume contamination from individual ROI masks (Figure 2). Each regional mean value was then extracted from the corrected ROI masks. The robust mean values were winsorized by excluding the ±5% of the regional extreme values. For R2*, *χ*, and qT1, the mean values were calculated from ROIs in T2*‐weighted space, while for CEST, they were calculated from downsampled masks in CEST space (Figure 2).

### 2.5 Immunohistochemical Processing and Quantification

After MRI, the animals were euthanized by cardiac perfusion with ice‐cold saline. The brains were removed, weighed, and photographed. Perfused brains were snap frozen in 2‐methylbutane submerged in dry‐ice for approximately 10 s before being stored in a −80°C freezer until use [63]. Brains were sliced coronally at a thickness of 25 μm across a 2‐mm slab, covering multiple regions of interest, including the hippocampus. Two sections were mounted per slide onto Superfrost Plus slides (Thermo Fisher) and stored at −80°C until use. The mounted sections were washed in phosphate buffered saline (PBS) for 10 min, followed by antigen retrieval solution using 1× Decloaker (Biocare Medical, RV1000) at 85°C for 10 min. Sections were blocked with 10% normal goat serum and 0.3% Triton X‐100 for 2 h, and then primary antibodies were incubated overnight. The antibodies included anti‐mouse amyloid‐beta N(6E10) for amyloid plaques, Prussian blue stain for iron deposition, and anti‐myelin basic protein (MBP) for myelin. Then, the sections were washed and further incubated with secondary antibodies Alexa‐fluor 488/555/647 for 2 h before mounting the section with an aqueous medium. Tile images were acquired using a 20× objective of a fluorescence microscope at a fixed exposure time and gain settings. Because of insufficient perfusion, one ARTE10 animal showed major staining artefacts and was excluded from further IHC‐based analysis.

To characterize hippocampal myelin levels, iron load, and Aβ deposition in the study animals, we used Qupath framework [64]. Qupath was initially used to manually draw ROIs (hippocampus) and segment stain‐specific patterns on a subset of each IHC stain. These manual annotations were then used to train stain‐specific machine learning models for automated quantification of ROI volumes and percentage of hippocampal volume covered by Aβ, iron, and MBP stains, respectively. For Prussian blue and MBP, we trained a pixel classifier based on random trees with default settings and hyperparameters, and for 6E10, we used a thresholding‐based classifier (Supplementary Figure 1).

### 2.6 Statistical Analysis

Statistical analysis was performed using linear mixed‐effects models (LMEMs) implemented in R (Version 4.3.3) with the “*lme4*” and “*lmerTest*” packages. Prior to the main statistical analysis, MRI data were residualized by regressing out ROI volume values to control for potential confounding effects of volumetric differences on qMRI measurements. This step was used to focus specifically on genotype effects and genotype × ROI interaction effects while controlling for the anatomical variability across different brain regions. The residualized values were then used in the LMEM models.

For each MRI index (R2*, *χ*, qT1, AREX_(3.5ppm)_, and AREX_(−3.5ppm)_), a separate LMEM was used with genotype (WT vs. ARTE10) and ROI (hippocampus, corpus callosum, thalamus, striatum) as fixed effects, including their interaction term to assess region‐specific pathological variations. Although ROI main effects were removed in the previous step (yielding *F* = 0 by design), ROI was retained in the model formula to enable testing of the (genotype × ROI) interaction, which assesses whether genotype effects differ across brain regions. To account for repeated measurements of four ROIs within each animal, random intercepts for individual animals were included in the model. Models were estimated using restricted maximum likelihood. Significance of fixed effects was assessed using type III *F* tests with Satterthwaite approximation for denominator degrees of freedom, with statistical significance set at *α* = 0.05 (uncorrected), treating each qMRI index as a separate analysis. Post hoc pairwise comparisons between genotypes within each ROI were conducted using estimated marginal means with Benjamini–Hochberg false discovery rate (FDR) correction for four comparisons per index. Effect sizes were estimated using Hedges' *g* and confidence intervals (CIs) for the effect size were estimated using cluster bootstrap resampling (1000 iterations) to preserve within‐animal correlation structure. Statistical significance was set at *α* = 0.05.

For IHC‐based group analyses, hippocampal IHC stain load (density) was calculated as a ratio of stain volume to the total hippocampal volume. Nonparametric tests were employed for all IHC‐based group comparisons due to unequal variances and/or zero‐inflated data in 6E10 (Aβ) stain. Normality was assessed using the Shapiro–Wilk test, and homogeneity of variance was evaluated using Levene's test. For IHC quantifications, the Mann–Whitney *U* test compared ARTE10 and WT groups when both groups had detectable IHC signal. The rank‐biserial correlation (*r*) was calculated as a nonparametric effect size measure, with |*r*| values of 0.1, 0.3, and 0.5 interpreted as small, medium, and large effects, respectively. For 6E10 stain of Aβ, wild‐type animals showed no detectable immunoreactivity; Fisher's exact test was used to compare the hippocampal volume of animals with detectable 6E10 signal between groups. Statistical significance was set at *α* = 0.05 (two‐tailed). Data are presented as median (Q1, Q3) for nonparametric analyses and mean ± standard deviation (SD) where appropriate.

We used correlation models to evaluate the relationship between MRI‐derived quantitative metrices and IHC stains in the hippocampus. The effect of hippocampal iron load on apparent transverse relaxation rate was assessed using partial correlation between hippocampal R2* and MBP, controlling for iron load. Furthermore, the effect of myelin/lipid on hippocampal magnetic susceptibility was tested using partial correlation between *χ* and hippocampal iron load, controlling for MBP density. *p* values < 0.05 were considered statistically significant.

## 3 Results

### 3.1 Effect of Aβ Pathology on Regional qMRI and IHC‐Derived Parameters

**FIGURE 4:**
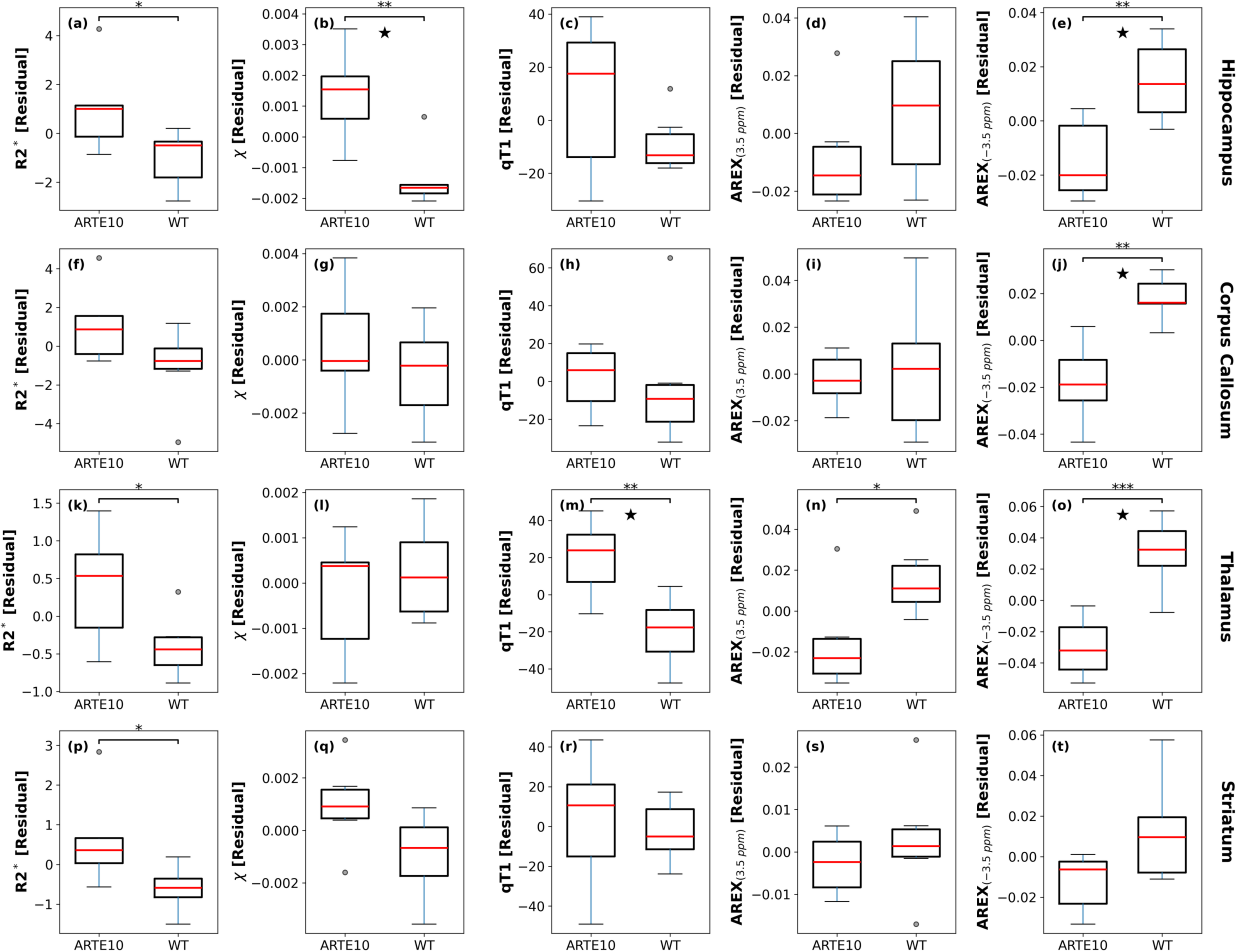
Boxplots illustrate group differences between mean hippocampal, corpus callosum, thalamus, and striatum qMRI‐ and CEST‐derived parametric map values in ARTE10 and WT mice. *n* = 6 per group. The analyses were adjusted for ROIs volumes. Significance: *p < 0.05, **p < 0.01, ***p < 0.001. Multiple comparisons across four regions of interest and five MRI indices were adjusted by false discovery rate (FDR) using the Benjamini–Hochberg criterion (*α* = 0.05), ^★^ *p*_FDR_ < 0.05. Effect sizes were estimated using Hedges' *g* and confidence intervals (CIs) for the effect size were estimated using cluster bootstrap resampling (1000 iterations).

Linear mixed‐effects model‐based analyses of residualized qMRI data showed significant group differences between ARTE10 and WT littermates across the selected ROIs, suggesting region‐specific vulnerability to Aβ pathology (Table 2). AREX_(−3.5ppm)_ showed a significant main effect of genotype (*F*(1,10) = 17.80; *p* < 0.01) and significant (genotype × ROI) interaction effect (*F*(3,30) = 6.46, *p* < 0.01), indicating that group differences varied substantially across the ROIs. qT1 showed a significant (genotype × ROI) interaction effect (*F*(3,30) = 5.39, *p* < 0.01), despite no significant main effect of genotype, suggesting region‐specific alterations. Additionally, *χ* and R2* showed significant main effects of genotype (*F*(1,10) = 7.077, *p* < 0.05 and *F*(1,10) = 8.101, *p* < 0.05, respectively), indicating overall group differences across selected regions. Post hoc pairwise comparison based on estimated marginal means with FDR correction showed that AREX_(−3.5ppm)_ was significantly lower in the thalamus (*p*_*FDR*_ = 0.01; Hedges' *g* = −2.64; CI [−4.59 to −1.62]), corpus callosum (*p*_*FDR*_ = 0.01; Hedges' *g* = −2.39; CI [−4.19 to −1.46]), and hippocampus (*p*_*FDR*_ = 0.03; Hedges' *g* = −1.81; CI [−3.20 to −0.95]) of ARTE10 animals, compared to the WT group (Table 3 and Figure 4). In the hippocampus, *χ* was significantly higher in ARTE10 group compared to the WT animals (*p*_*FDR*_ = 0.02; Hedges' *g* = 1.99; CI [1.07 to 4.75]), and in the thalamus, qT1 was significantly higher in ARTE10 animals compared to the WT group (*p*_*FDR*_ = 0.03; Hedges' *g* = 1.84; CI [1.00 to 3.16]). R2* demonstrated consistently large effect sizes across all examined ROIs (Hedges' *g* = 0.98–1.22), with a significant increase in the hippocampus (*p* = 0.044), thalamus (*p* = 0.048), and the striatum (*p* = 0.045), although these comparisons did not survive FDR correction, potentially reflecting limited statistical power given the sample size. The regional mean and standard deviation values of qMRI‐ and CEST‐derived metrics are provided in the supplementary Table 1.

**TABLE 2:** Comparison of MRI‐derived metrices in the selected ROIs across groups.

Effect	*p*	*F*	df	df error
R2* (1/s)				
Group	0.017*	8.101	1	10
Interaction (group × ROI)	0.437	0.930	3	30
***χ* (ppm)**				
Group	0.023*	7.077	1	10
Interaction (group × ROI)	0.095	2.318	3	30
**qT1 (ms)**				
Group	0.214	1.758	1	10
Interaction (group × ROI)	0.004**	5.393	3	30
**AREX**_**(3.5ppm)**_				
Group	0.125	2.792	1	10
Interaction (group × ROI)	0.115	2.146	3	30
**AREX**_**(−3.5ppm)**_				
Group	0.002**	17.80	1	10
Interaction (group × ROI)	0.001**	6.464	3	30

*Note:* Data were residualized by regressing out ROI‐volumes prior to analysis; therefore, ROI main effects are not shown (*F* = 0 by design).

Abbreviations: AREX, apparent exchange‐dependent relaxation; qT1, quantitative T1; R2*, apparent transverse relaxation rate.

Significance: *p < 0.05, **p < 0.01.

**TABLE 3:** Pair‐wise group differences with the post hoc analyses.

ROIs	MRI indices	Mean difference (ARTE10‐WT)	p	Effect size (Hedges' *g*)	Bootstrap 95% CI
Hippocampus	R2*	2.028	0.044*	1.220	0.5449 to 1.9022
	*χ*	0.0027	0.003**	1.994	1.0722 to 4.7579
	qT1	17.429	0.199	0.731	−0.0623 to 3.11
	AREX_(3.5ppm)_	−0.016	0.227	−0.685	−2.4026 to 0.0308
	AREX_(−3.5ppm)_	−0.024	0.006**	−1.811	−3.2019 to −0.9529
Corpus callosum	R2*	2.169	0.094	0.985	0.2869 to 1.5651
	*χ*	0.0009	0.457	0.412	−0.4134 to 1.22
	qT1	3.407	0.834	0.114	−0.5007 to 1.8162
	AREX_(3.5ppm)_	−0.005	0.713	−0.201	−1.1316 to 0.828
	AREX_(−3.5ppm)_	−0.035	0.001**	−2.396	−4.1973 to −1.4682
Thalamus	R2*	0.798	0.048*	1.196	0.3896 to 2.5671
	*χ*	−0.0005	0.499	−0.373	−1.056 to 0.5213
	qT1	39.607	0.006**	1.840	1.0062 to 3.1624
	AREX_(3.5ppm)_	−0.031	0.030*	−1.336	−3.2886 to −0.5581
	AREX_(−3.5ppm)_	−0.060	0.0005***	−2.641	−4.5923 to −1.6189
Striatum	R2*	1.224548333	0.045*	1.217	0.6938 to 2.0344
	*χ*	0.001915	0.071	1.076	0.354 to 1.9575
	qT1	5.60848	0.717	0.198	−0.6317 to 1.397
	AREX_(3.5ppm)_	−0.005576667	0.407	−0.460	−1.3232 to 0.3802
	AREX_(−3.5ppm)_	−0.024916667	0.066	−1.095	−1.7838 to −0.4979

*Note:* Analyses adjusted for ROIs volumes. Multiple comparisons across four regions of interest and five MRI indices were adjusted for false discovery rate (FDR) using the Benjamini–Hochberg criterion (*α* = 0.05), *p*_FDR_ < 0.05. *p* values in bold survived FDR correction for multiple comparison. Effect sizes were estimated using Hedges' *g* and confidence intervals (CIs) for the effect size were estimated using cluster bootstrap resampling (1000 iterations).

Significance: *p < 0.05, **p < 0.01, ***p < 0.001.

**FIGURE 5:**
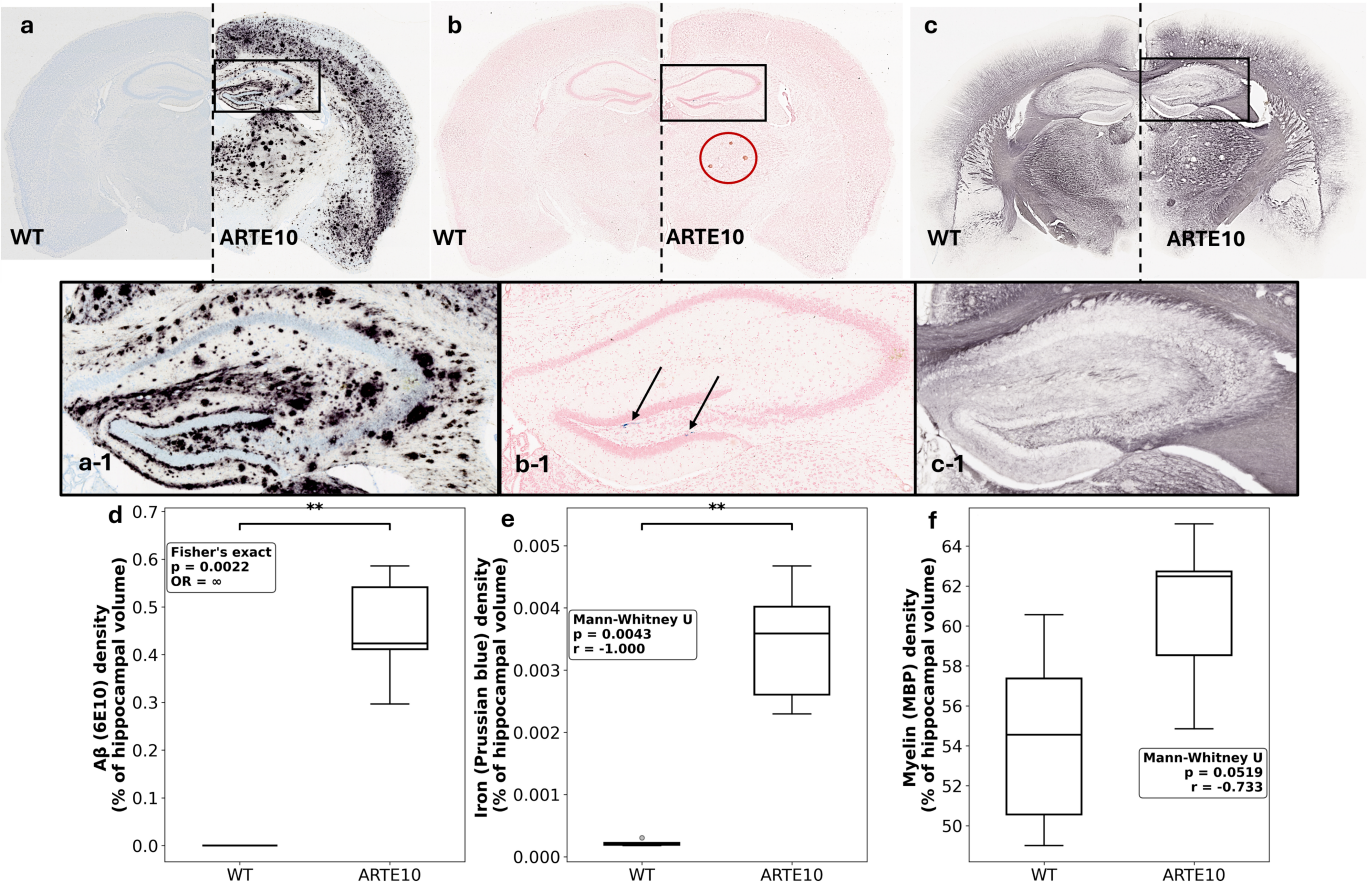
Immunohistochemical staining images for (a) Aβ plaque load using 6E10 stain, (b) Prussian blue stain to identify cerebral microbleeds, and (c) MBP to quantify myelin composition. The stains are from representative WT and ARTE10 animals. Insets a‐1, b‐1, and c‐1, depict the hippocampal region of ARTE10 animal. In b, congophilic dense‐core plaques are visible in the thalamic region (dark red circle) and in b‐1 small regions of microbleeds are also identifiable in the hippocampal region (black arrows). The boxplots show the group differences in hippocampal Aβ load (d), iron load (e), and myelin density (f). Five ARTE10 animals, and six WT mice were used for this group comparison. Mann–Whitney *U* test compared ARTE10 and WT groups for iron and myelin stains. The rank‐biserial correlation (*r*) was calculated as a nonparametric effect size measure, with |*r*| values of 0.1, 0.3, and 0.5 interpreted as small, medium, and large effects, respectively. For 6E10 stain of Aβ, wild‐type animals showed no detectable immunoreactivity, Fisher's exact test was used to compare the proportion of animals with detectable 6E10 signal between groups. Statistical significance was set at *α* = 0.05 (two‐tailed). OR, odds ratio.

Aβ burden was quantified in hippocampal sections using immunohistochemistry. No Aβ (6E10) immunoreactivity was detected in any WT animal (0/6, 0%), while all ARTE10 mice showed robust Aβ deposition in the hippocampus (5/5, 100%; Fisher's exact test, *p* = 0.002). In ARTE10 mice, Aβ burden was 0.45% ± 0.12% of the measured hippocampal volume (mean ± SD; median 0.42% (interquartile range [IQR]: 0.41%–0.54%]; range 0.29–0.58). Using Prussian blue stains, the hippocampal iron load was significantly higher in ARTE10 mice compared to the WT animals (Figure 5). ARTE10 animals showed significantly higher iron burden (median: 0.0036% [IQR: 0.0026%–0.0040%]) compared to WT mice (median: 0.00022% [IQR: 0.00020%–0.00024%]; Mann–Whitney *U* test: *U* = 30.00, *p* = 0.0043, rank‐biserial *r* = −1.000), representing a 16.3‐fold increase in hippocampal iron load. The complete separation between groups (*r* = −1.000) indicated no overlap in iron levels. MBP immunoreactivity showed a trend toward increased staining signal in ARTE10 mice compared to WT controls. ARTE10 mice showed a median MBP density of 62.48% (IQR: 58.54%–62.73%) compared to 54.56% (IQR: 50.57%–57.37%) in WT mice (Mann–Whitney *U* test: *U* = 26.00, *p* = 0.052, rank‐biserial *r* = −0.733), representing a 1.15‐fold difference.

### 3.2 Correlation of Hippocampal IHC Measurements With MRI Parameters

**FIGURE 6:**
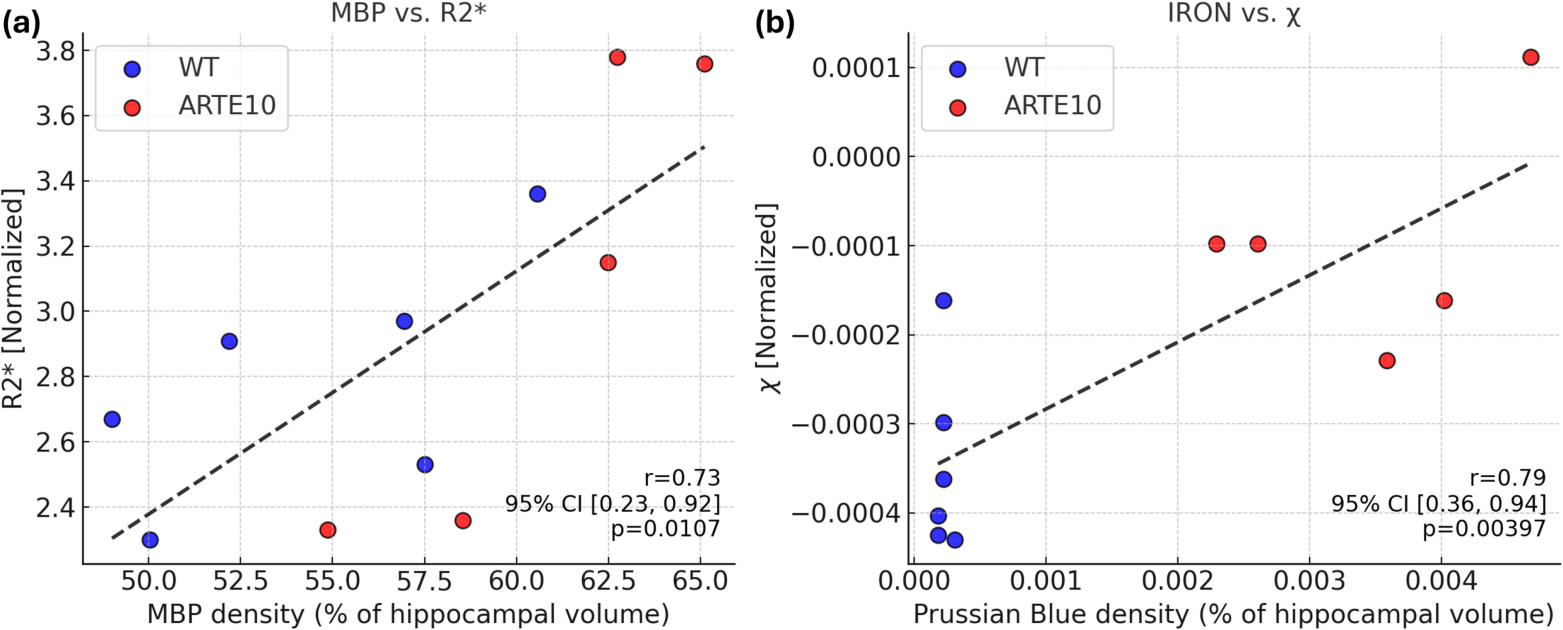
(a) Correlation between hippocampal MBP density and apparent transverse relaxation (R2*). (b) Correlation between hippocampal iron load using Prussian blue stain and regional magnetic susceptibility (*χ*).

Table 4 and Figure 6 show the correlation between the hippocampal IHC measurements and MRI‐derived quantitative parameters. Significant positive association was observed between hippocampal MBP density and R2* (Pearson's correlation *r* = 0.73; *p* < 0.05) and between MBP density and hippocampal qT1 (*r* = 0.69; *p* < 0.05). Strong positive correlation was also observed between hippocampal iron load and magnetic susceptibility (*r* = 0.79; *p* < 0.01). The partial correlation between MBP density and R2* remained significant after controlling for the effect of iron load (*r* = 0.81; *p* < 0.01). Similarly, after controlling for the effect of myelin load, the partial correlation between iron load and *χ* exhibited significant positive association (*r* = 0.73, *p* < 0.05).

**TABLE 4:** Correlation between quantitative MRI parameters and IHC‐derived parameters.

MRI parameters	MBP density (% of hippocampal volume), *n* = 11		Prussian blue (iron) density (% of hippocampal volume), *n* = 11		6E10 (Aβ) density (% of hippocampal volume), *n* = 5	
	*r*	*p*	*r*	*p*	*r*	*p*
R2*	0.73	**0.01** ^**†**^	0.07	0.83	0.45	0.44
*χ*	0.43	0.18	0.79	**0.004** ^**†**^	−0.58	0.30
qT1	0.69	**0.02** ^**†**^	−0.04	0.91	0.12	0.84
AREX_(−3.5ppm)_	0.44	0.17	−0.10	0.77	0.25	0.68
AREX_(3.5ppm)_	0.43	0.18	−0.16	0.63	0.39	0.52
AREX_(2.0ppm)_	0.42	0.19	0.18	0.60	0.17	0.78

^†^ *p* < 0.05.

## 4 Discussion

In this study, we applied a multimodal MR approach to map regional myelin/lipid variations and iron load (cerebral microbleeds) in a 10‐month‐old murine model of Aβ pathology. To validate our in vivo MR findings, we also performed IHC staining sensitive to amyloid, iron, and myelin in the same cohort of animals. Our LMEM‐based analyses showed significant genotype‐related changes in multiple MRI parameters across the four a priori ROIs, suggesting differential regional vulnerability to Aβ pathology (Tables 2 and 3 and Figure 4). AREX_(−3.5ppm)_ showed significant main effect of genotype and significant interaction effect (genotype × ROI). Post hoc comparisons with FDR correction revealed significant AREX _(−3.5 ppm)_ reductions in ARTE10 mice across the hippocampus, corpus callosum, and thalamus, with the thalamus exhibiting the most pronounced effect. qT1 showed a significant (genotype × ROI) interaction with region‐specific elevation in the thalamus of ARTE10 animals, suggesting region‐specific pathological involvement. *χ* exhibited a significant main effect of genotype with significant increase in the hippocampus of ARTE10 mice, consistent with increased magnetic susceptibility reflecting iron accumulation/microbleeds. R2* showed a significant main effect of genotype with large effect sizes across multiple ROIs. However, individual post hoc comparisons did not survive FDR correction, suggesting limited statistical power with *n* = 6 per group rather than absence of pathophysiological effects. To estimate group‐wise changes in hippocampal Aβ load, myelin composition, and iron load (microbleeds), we used IHC‐derived 6E10, MBP, and Prussian blue stains (Figure 5a–c). We observed robust Aβ plaque deposition in the hippocampus of ARTE10 mice, and hippocampal iron load was also significantly higher in the ARTE10 group, compared to the WT littermate (Figure 5d,c). Hippocampal MBP density was not statistically different between the groups, despite showing large effect size (Figure 5f). Correlation analyses showed a strong positive association between hippocampal R2* and IHC‐derived MBP, irrespective of the genotype, which remained significant after controlling for the effect of iron load. In addition, a positive association between hippocampal *χ* and IHC‐derived iron load remained significant, ever after controlling for the effect of MBP. These patterns of region‐specific vulnerability may reflect differential influence of pathological processes, regional variations in cellular composition, or heterogeneous involvement of Aβ‐instigated mechanisms such as neuroinflammation, iron dysregulation, or myelin alterations.

These regional differences in apparent relaxation rate, bulk magnetic susceptibility, T1 relaxation time, and AREX are in general agreement with previous studies on the animal models of neurodegeneration [12, 31, 65, 66, 67]. Region‐specific alterations in R2*, *χ*, qT1, and rNOE_(−3.5)_ may suggest relatively lower levels of lipid or macromolecular concentration in the hippocampus as compared to the corpus callosum or thalamus. Aβ and myelin are diamagnetic and cause the reduction in T2* decay. Our results and previous studies also support the impact of myelin diamagnetic nature on MR signal perturbation and how regional changes in myelin can influence R2* and bulk magnetic susceptibility measurement [65, 68]. Some of the contradictory results in these aforementioned studies could be due to the specific strain of transgenic models, magnetic field strength (4.7, 7, 9.4, or 11.7 T etc.), MR protocol parameters, in vivo versus ex vivo paradigm, and age/sex of animals. For example, we used male ARTE10 mice, which are double (APP/PS1) transgenic model of AD. This model develops cerebral β‐amyloidosis and with advanced plaque load also exhibits vascular Aβ deposition. Other studies have either used APP^NL‐F^ knock‐in and 5xFAD model of Aβ or tau models of neurodegeneration [13, 67], which provide unique aspects of neurodegeneration but do not capture the combined effects of heterogeneous pathological mechanisms associated with cerebral and vascular Aβ accumulation.

Aβ protein has metal ion binding sites, which facilitates iron and ferritin storage within amyloid plaques [9] causing oxidative stress [69], which further exacerbates toxic protein (Aβ peptide) aggregation [34, 70, 71]. The accumulation of endogenous iron deposition within and around Aβ plaques can lead to further heterogeneity in regional apparent relaxation rate and bulk magnetic susceptibility measurements [72]. The Aβ and iron interplay was observed in our study (Figure 5 and Table 4). We observed higher R2* and *χ* in the hippocampus of ARTE10 animals compared to WT animals, while other regions including corpus callosum showed no significant changes, suggesting region‐specific iron accumulation patterns in the ARTE10 model. This hippocampus‐specific *χ* elevation is corroborated by our histological findings showing significantly higher Prussian blue staining in ARTE10 hippocampus (Figure 5b) and the strong correlation between *χ* and iron density (Table 4). These results are also supported by previous studies [31, 65], thus highlighting the role of iron deposition as an important factor in Aβ toxicity.

In our study, focal regions of higher R2* were visually observed in the hippocampus and thalamic regions of ARTE10 mice (Figure 1b). R2* relaxation rate is influenced by a number of physiological factors such as iron deposition, deoxyhemoglobin, blood/tissue oxygen levels, lipid/myelin composition, calcification, vascular and capillary density, and vascular and parenchymal Aβ load [65, 73, 74, 75, 76, 77, 78]. Therefore, it is difficult to isolate diamagnetic effects from iron‐induced perturbation solely by using R2*/T2* maps. Unlike R2* maps, which showed limited discrimination between iron load and other pathological processes in the thalamic region, *χ* maps showed high contrast between regions of positive and negative susceptibilities in the hippocampus (microbleeds) as well as in the thalamic regions (congophilic dense‐core plaques) (Figures 1b and 5b,b‐1). The absence of iron deposition/microbleeds in the thalamic region were further confirmed from the Prussian blue stains (Figure 5). Because Prussian blue stain selectively binds to ferric iron (Fe^3+^), these observations suggest some other pathological processes, involving different forms of iron, astrogliosis around diffused plaques, lipid variation, impairment of membrane integrity, or perhaps points toward the vascular remodeling or compromised blood brain barrier as a result of vascular Aβ insult [79, 80]. Using AβPPswe/PS1dE9 model of AD, it has been suggested that increase in hippocampal and cortical qT1 is associated with astrogliosis. However, the authors of the study did not find significant qT1 changes in the thalamus [81]. Using Tg2576 mouse model of AD, Kumar, Yang [82] reported significant (age × genotype) interaction effects on qT1 in the thalamus and hypothalamus and genotype effects for isocortex and hippocampus. These studies attribute qT1‐associated changes to the water accumulation associated with amyloidosis and iron load [83].

Cholesterol and phospholipid have been implicated in lipid homeostasis and vessel wall structural health [84, 85]. Increased phospholipid loss suggests myelin breakdown and has also been associated with AD [86, 87, 88]. We observed a trend toward elevated hippocampal MBP density in ARTE10 compared to WT (Figure 5f). This finding may suggest a compensatory myelin response or altered myelin protein to lipid composition rather than overall myelin loss. This observation is consistent with recent literature suggesting regional increase in MBP but impaired myelin ultrastructure and function during the early stages of the pathology [8, 89]. The myelin debris can overwhelm the brain waste clearance system, reducing the microglial ability to phagocytose pathological Aβ aggregates, thus further accelerating amyloidosis [90]. Additionally, Aβ mediated iron deposition can overdrive iron‐catalyzed lipid peroxidation, leading to cellular membrane damage [91]. Phospholipid breakdown can affect the production and conversion of soluble Aβ_40_ into fibrillar Aβ aggregates leading to CAA [35]. Recent studies have attributed membrane lipids (aliphatic chains of phospholipids) as a primary source of NOE_(−3.5 ppm)_ signal in the brain [67, 92]. Region‐wise variation in NOE_(−3.5 ppm)_ signal has been attributed to tissue‐specific composition of mobile phospholipids [28]. Our CEST‐based results also showed reduced AREX _(−3.5 ppm)_ in the hippocampus, corpus callosum, and thalamus of ARTE10 animals, compared to the WT controls. Additionally, we also observed region‐specific variations in NOE _(−3.5 ppm)_ signal, irrespective of the genotype. We did not observe an association between hippocampal AREX_(−3.5ppm)_ and IHC‐derived hippocampal MBP density, but we did observe a positive association between qT1 and MBP density, which may indicate that NOE_(−3.5 ppm)_ is not sensitive to the myelin sheath protein marker (MBP), but to myelin lipids [28].

The APT‐based AREX_(3.5ppm)_ showed a reduction in the thalamus of ARTE10 mice compared to the WT group (Table 3 and Figure 4). Although this comparison did not survive the FDR‐based correction for multiple comparisons, our AREX_(3.5ppm)_‐based findings align with previous APT studies in the 5xFAD mouse model of AD [93]. The robust genotype effects observed for AREX _(−3.5 ppm)_ compared to AREX _(3.5 ppm)_ likely reflect both the greater sensitivity of rNOE‐based contrast to lipid/phospholipid alterations and our acquisition optimization for the rNOE signal.

This study has some limitations. Because of the exploratory nature of the work, we opted for a cross‐sectional study design (10‐month‐old male mice) representing established cerebral and vascular amyloidosis. Our analysis is focused on four a priori brain regions (the hippocampus, corpus callosum, thalamus, and striatum) selected for their relevance to AD pathology. Future longitudinal studies should include sex as a biological variable and examine additional brain regions, particularly the cerebral cortex. The cortex comprises multiple structurally and functionally distinct regions, each with unique cytoarchitectural organization and connectivity patterns. The heterogeneous cortical tissue composition may pose differential vulnerability to Aβ pathology. Incorporating multiple cortical regions into the current study would necessitate additional multiple comparison corrections, substantially reducing the statistical power. With *n* = 6 per group, the study was adequately powered to detect large effects (Hedges' *g* ≥ 0.8) across four ROIs, but extending this approach to include multiple cortical regions would further limit the statistical power of the current study. Comprehensive cortical characterization should therefore be pursued in future studies with larger sample sizes, enabling robust detection of region‐specific effects while maintaining appropriate control over type I error. While IHC‐based validation was performed for the hippocampus, we did not obtain histological quantification for thalamus, corpus callosum, or striatum. Therefore, interpretation of MRI findings in these regions relied on the established biophysical basis of the MRI contrast mechanisms and inference from the existing literature on regional AD pathology. In this study, we did not explore the effect of susceptibility anisotropy on regional *χ* quantification [94]. These effects would be more pronounced in corpus callosum and warrant further investigation [95, 96].

## 5 Conclusion

In this study, we investigated the effect of Aβ pathology on regional myeloarchitectural integrity using qMRI, CEST‐MRI, and IHC. This multimodal approach allowed us to deploy multiple intrinsic MR mechanisms/contrasts affected by cerebral and vascular Aβ deposition. 3D qMRI and 2D multislice CEST imaging mapped the myeloarchitectural variations and microbleed load across the entire hippocampus, corpus callosum, thalamus, and striatum. High‐resolution QSM separated myelin/diamagnetic substrates from hemosiderin deposits, providing improved assessment of regional iron load. CEST‐derived AREX _(−3.5 ppm)_ contrast specifically enabled assessment of regional phospholipid alterations in relation to cerebral and vascular Aβ burden. Additionally, multislice IHC stains for Aβ, iron, and MBP promoted a better understanding of the physiological mechanism responsible for MRI‐derived signal perturbations. This multimodal neuroimaging framework can improve our understanding of various pathological processes in AD, highlighting its potential for improved early detection, therapeutic monitoring, and development of targeted interventions.

## Author Contributions

Conceptualization: Syed Salman Shahid and Yu‐Chien Wu. Data curation: Syed Salman Shahid, Erin E. Jarvis, Xuan Li, and Aibo Wang. Formal analysis: Syed Salman Shahid and Yu‐Chien Wu. Funding acquisition: Syed Salman Shahid and Yu‐Chien Wu. Investigation: Syed Salman Shahid, Xuan Li, Yomna Takieldeen, Aibo Wang, and Yu‐Chien Wu. Methodology: Syed Salman Shahid, Xuan Li, Mario Dzemidzic, and Yu‐Chien Wu. Project administration: Erin E. Jarvis and Yu‐Chien Wu. Resources: Syed Salman Shahid and Yu‐Chien Wu. Software: Syed Salman Shahid and Xuan Li. Supervision: Yu‐Chien Wu. Validation: Syed Salman Shahid and Yu‐Chien Wu. Visualization: Syed Salman Shahid and Xuan Li. Writing – original draft: Syed Salman Shahid and Mario Dzemidzic. Writing – review and editing: Syed Salman Shahid, Mario Dzemidzic, Erin E. Jarvis, Donna Wilcock, and Yu‐Chien Wu.

## Funding

This work was supported in part by Indiana Alzheimer’s Disease Research Center Development Project Program (SSS), Ralph W. and Grace M. Showalter Research Trust (SSS), Roberts Neuroscience Imaging Research Fund, Stark Neurosciences Research Institute, Indiana University (SSS), National Institutes of Health R01AG083951 (YCW). The funders had no role in the study design, data collection and analysis, decision to publish or preparation of the manuscript.

## Supporting information


**Figure S1:** QuPath‐based IHC quantification. (A) Extensive Aβ plaque deposition throughout brain parenchyma of an ARTE10 mouse at 10 months using 6E10 stain. (a‐1) The sections were traced, and plaques were automatically outlined by the machine learning model trained in QuPath. (a‐2) Binary mask of the segmented plaque area. (b and b‐1) 6E10 stain on a 10‐month‐old WT animal processed and analyzed in the same manner.
**Table S1:**: Regional mean and SD values of qMRI‐ and CEST‐derived metrics. *n* = 6 per group; values are represented as mean ± standard deviation.

## Data Availability

The datasets used and/or analyzed during the current study are available from the corresponding author on reasonable request.
