# Supplementary material for: Multimodal MRI Reveals Cerebral and Vascular Amyloid‐Driven Myeloarchitectural Disorganization in a Mouse Model of Alzheimer's Disease
Source: NMR Biomed. 2026 Mar 13;39(4):e70262. doi: 10.1002/nbm.70262 (PMC12983127; doi:10.1002/nbm.70262)
Supplement: Supplementary file 1 — Figure S1: QuPath‐based IHC quantification. (A) Extensive Aβ plaque deposition throughout brain parenchyma of an ARTE10 mouse at 10 months using 6E10 stain. (a‐1) The sections were traced, and plaques were automatically outlined by the machine learning model trained in QuPath. (a‐2) Binary mask of the segmented plaque area. (b and b‐1) 6E10 stain on a 10‐month‐old WT animal processed and analyzed in the same manner. Table S1:: Regional mean and SD values of qMRI‐ and CEST‐derived metrics. n = 6 per group; values are represented as mean ± standard deviation. [file NBM-39-e70262-s001.docx]

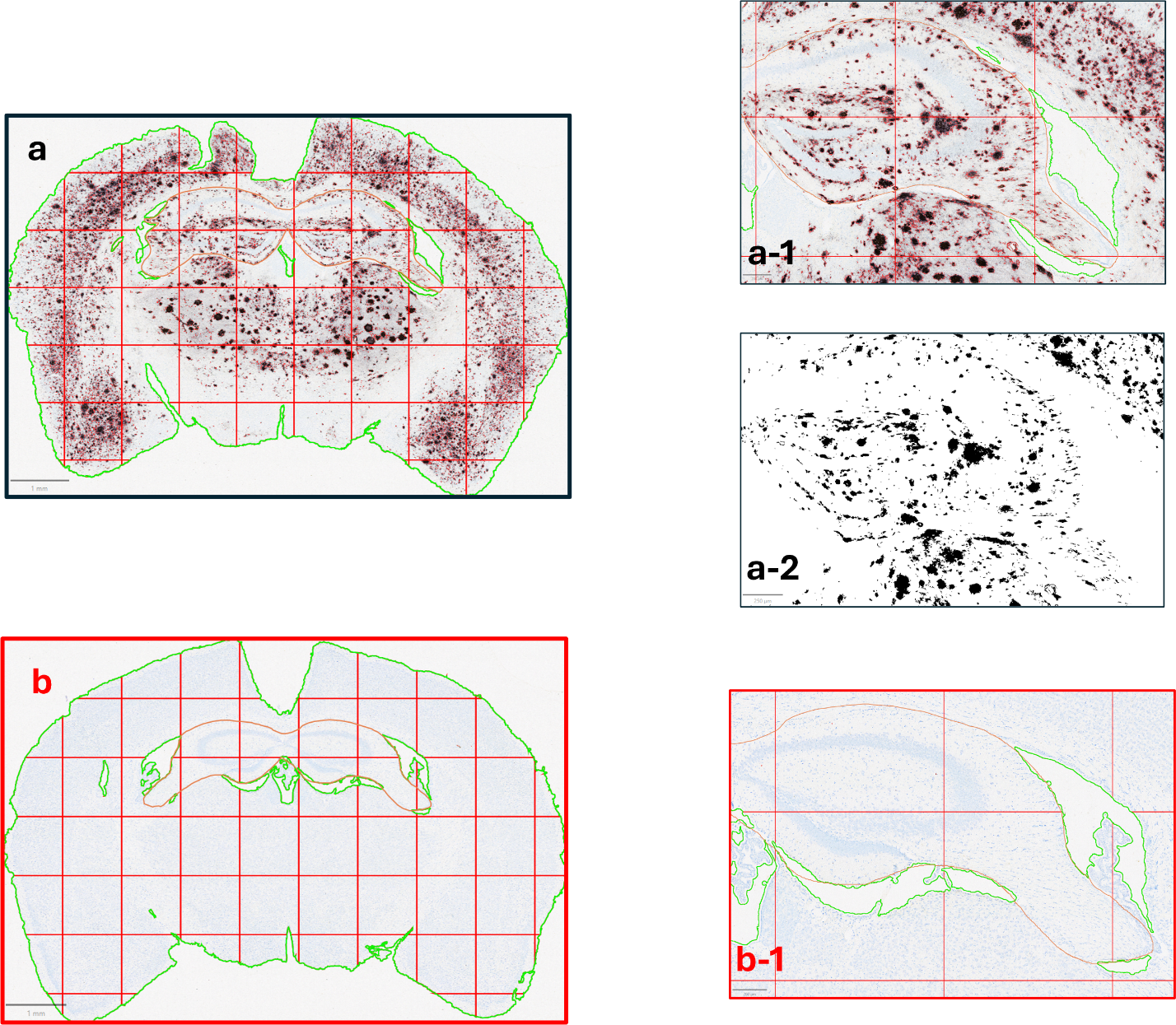


**Supplementary Figure 1**: QuPath based IHC quantification. A) extensive Aβ plaque deposition throughout brain parenchyma of an ARTE10 mouse at 10 months using 6E10 stain. (a-1), the sections were traced, and plaques were automatically outlined by the machine learning model trained in QuPath. (a-2), binary mask of the segmented plaque area. (b and b-1), show 6E10 stain on a 10-month-old WT animal processed and analyzed in the same manner.

**Supplementary Table 1**: Regional mean and SD values of qMRI and CEST derived metrics. *n*=6 per group; values are represented as mean ± standard deviation.

| **parameters** | **WT** | **ARTE10** |
| --- | --- | --- |
| **hippocampus** | | |
| **R2*(s^-1^)** | 29.84 ± 1.28 | 31.63 ±2.31 |
| **χ (ppm)** | -0.0037 ± 0.011 | -0.00096 ± 0.0014 |
| **AREX_(-3.5ppm)_ (s^-1^)** | 0.34 ± 0.012 | 0.312 ± 0.019 |
| **T1 (ms)** | 1704.10 ± 15.81 | 1725.16 ± 29.36 |
| **AREX_(3.5ppm)_ (s^-1^)** | 0.10±0.02 | 0.08±0.02 |
| **AREX_(2.0ppm)_ (s^-1^)** | 0.17±0.03 | 0.19±0.01 |
| **Corpus callosum** | | |
| **R2*(s^-1^)** | 41.10 ± 1.89 | 43.36 ± 2.14 |
| **χ (ppm)** | -0.021 ± 0.002 | -0.021 ± 0.002 |
| **AREX_(-3.5ppm)_ (s^-1^)** | 0.43 ± 0.009 | 0.394 ± 0.017 |
| **T1 (ms)** | 1521 ± 35.06 | 1525 ± 16.98 |
| **AREX_(3.5ppm)_ (s^-1^)** | 0.10±0.03 | 0.10±0.01 |
| **AREX_(2.0ppm)_ (s^-1^)** | 0.22±0.04 | 0.24±0.02 |
| **Striatum** | | |
| **R2*(s^-1^)** | 33.06 ± 0.57 | 34.37±1.15 |
| **χ (ppm)** | -0.002±0.001 | -0.0002±0.001 |
| **AREX_(-3.5ppm)_ (s^-1^)** | 0.43±0.03 | 0.32±0.01 |
| **T1 (ms)** | 1685.95±15.62 | 1691.72±33.46 |
| **AREX_(3.5ppm)_ (s^-1^)** | 0.98±0.01 | 0.08±0.007 |
| **AREX_(2.0ppm)_ (s^-1^)** | 0.17±0.03 | 0.18±0.01 |
| **Thalamus** | | |
| **R2*(s^-1^)** | 33.03±0.43 | 43.04±0.97 |
| **χ (ppm)** | 0.005±0.001 | 0.004±0.001 |
| **AREX_(-3.5ppm)_ (s^-1^)** | 0.41±0.03 | 0.35±0.02 |
| **T1 (ms)** | 1607.47±17.67 | 1648.24±20.91 |
| **AREX_(3.5ppm)_ (s^-1^)** | 0.13±0.021 | 0.10±0.02 |
| **AREX_(2.0ppm)_ (s^-1^)** | 0.19±0.02 | 0.21±0.01 |
